# Secondary Bacterial Infections in Critically Ill COVID-19 Patients Admitted in the Intensive Care Unit of a Tertiary Hospital in Romania

**DOI:** 10.3390/jcm13206201

**Published:** 2024-10-18

**Authors:** Ionela-Anca Pintea-Simon, Ligia Bancu, Anca Delia Mare, Cristina Nicoleta Ciurea, Felicia Toma, Mădălina Cristina Brukner, Anca-Meda Văsieșiu, Adrian Man

**Affiliations:** 1 Doctoral School of Medicine and Pharmacy, George Emil Palade University of Medicine, Pharmacy, Science and Technology of Târgu Mures, 540142 Targu Mures, Romania; ionela.pintea-simon@umfst.ro; 2 Department of Internal Medicine M3, George Emil Palade University of Medicine, Pharmacy, Science and Technology of Târgu Mures, 540142 Targu Mures, Romania; 3 Department of Microbiology, George Emil Palade University of Medicine, Pharmacy, Science and Technology of Târgu Mures, 540142 Targu Mures, Romania; anca.mare@umfst.ro (A.D.M.); cristina.ciurea@umfst.ro (C.N.C.); felicia.toma@umfst.ro (F.T.); adrian.man@umfst.ro (A.M.); 4 Department of Infectious Disease, George Emil Palade University of Medicine, Pharmacy, Science and Technology of Târgu Mures, 540142 Targu Mures, Romania; cristina.brukner@yahoo.com (M.C.B.); anca-meda.vasiesiu@umfst.ro (A.-M.V.)

**Keywords:** antibiotic resistance, SARS-CoV-2, pneumonia

## Abstract

**Background:** The outbreak of the COVID-19 pandemic caught healthcare systems in many countries unprepared. Shortages of personnel, medicines, disinfectants, and intensive care unit (ICU) capacities, combined with inadvertent use of antibiotics and emergence of drug-resistant secondary infections, led to a surge in COVID-19-related mortality. **Objective:** We aimed to evaluate the prevalence of secondary bacterial infections and the associated antibiotic resistance in a temporary established ICU dedicated to COVID-19 patients. We also assessed the utility of clinical and routine laboratory data as predictors of secondary infections and mortality in these patients. **Methods:** We examined the medical records of 243 patients admitted to the COVID-19 Medical Support Unit of Târgu Mures, Romania, between 1 August 2020 and 31 January 2021. **Results:** Among the 243 patients admitted to the COVID-19 Medical Support Unit of Târgu Mures between 1 August 2020 and 31 January 2021, 59 (24.3%) presented secondary infections. *Acinetobacter baumannii* and *Klebsiella pneumoniae* were the most frequent isolates (31.1% and 18.9%, respectively), most of them multidrug resistant. Chronic obstructive pulmonary disease had a higher prevalence in patients who developed secondary infections (*p* = 0.012). Secondary infections were associated with longer stay in the ICU and with higher mortality (*p* = 0.006 and *p* = 0.038, respectively). **Conclusions:** Early identification of secondary infections and proper use of antibiotics are necessary to limit the spread of multidrug-resistant microorganisms in COVID-19 patients admitted in the ICU.

## 1. Introduction

The coronavirus disease 2019 (COVID-19), caused by SARS-CoV-2 infection, was first described five years ago in Wuhan, China. Starting with apparently few epidemiologically linked cases of pneumonia, the disease rapidly evolved into a pandemic that swiped the world in several successive waves. COVID-19 has a wide range of manifestations, from asymptomatic forms to severe viral pneumonia evolving to respiratory failure, multiple organ dysfunction syndrome, sepsis, and septic shock [1].

Comorbidities and co-infection, especially in elder patients, augment the risk for severe forms of the disease [2]. Death is precipitated by single or multiple organ failure. Patients who develop acute respiratory distress syndrome, heart or renal failure, shock, or liver injury have poor outcomes [3].

Secondary bacterial infections often emerge in relationship with invasive mechanical ventilation and respiratory viral diseases. In COVID-19, altered immune response and lung injury due to SARS-CoV-2 favorize microbial colonization in hospitalized patients. COVID-19 is a risk factor for the development of secondary bacterial pneumonia [4]. Half of the patients who died during hospitalization for COVID-19-associated conditions had developed secondary bacterial infections, as reported in a large multicentric study [2].

The co-occurrence of bacterial or viral infections with acute respiratory failure related to SARS-CoV-2 pneumonia in patients admitted to intensive care units (ICUs) is poorly studied [3,5]. The epidemiology of bacterial infections associated with COVID-19 widely varies with setting and geographic region, as well as with time. An early report indicated that, among 17 patients admitted to a North American intensive care unit, 41% developed co-infections [6]. A meta-analysis based mostly on data from Chinese centers found the prevalence of bacterial co-infections and secondary infections in individuals hospitalized for COVID-19 to be rather low, around 3.5% to 14.3% [7]. A more recent meta-analysis that included studies from more than 40 countries estimated the pooled prevalence of bacterial co-infections to be 5.3% and that of secondary infections to be 18.4%. The prevalence of both types of infection in Europe seemed to be higher than in the Americas and lower than in the Eastern Mediterranean region. Per-organism antibiotic resistance in Europe was, however, lower than in most other regions [8]. After the first waves of the pandemic, both the rate of secondary bacterial infections related to COVID-19 and that of the antibiotic resistance showed a descendant trend [9].

Antibiotic resistance is a major threat for public health, and its manifestation considerably increased during the COVID-19 pandemic [10]. Among other factors, widespread and unnecessary use of antibiotics fueled a vicious cycle leading to antimicrobial resistance and the emergence and spread of resistant pathogens [10].

The discordance between the high frequency of antibiotic prescription and the relatively low prevalence of bacterial infections highlights a potential overuse of antibiotics in these patients. Antibiotic overuse in COVID-19 patients may lead to increased selective pressure for antimicrobial resistance [11]. Unnecessary use of antibiotics heightens the risk of antibacterial resistance, adverse events, and *Clostridioides difficile* infection [11]. Although COVID-19 is a viral disease and the initial estimates suggested that few COVID-19 patients presented bacterial co-infection, various antibiotics were recommended for use in the management of COVID-19, with some variability among geographic regions. For example, cephalosporins and carbapenems were recommended by the National Treatment Guidelines in 10 African countries [10]. A large cohort study of almost 65,000 American patients showed that 76% of individuals hospitalized with COVID-19 received at least one antibiotic. One third of them were treated with an anti-Pseudomonas drug, whereas another third received antibiotics active against MRSA. Ceftriaxone, azithromycin, and vancomycin were the most frequently prescribed antibiotics [11].

*Klebsiella pneumoniae*, *Acinetobacter baumannii*, and *Pseudomonas aeruginosa* are often identified as etiological agents of co-infections in COVID-19 patients, leading to exacerbation of the disease. Carbapenem-producing *Enterobacteriaceae* (CPE), carbapenem-resistant *Acinetobacter baumannii* (CRAB), and *Pseudomonas aeruginosa* (CRPA) are the most commonly isolated pathogens among patients with underlying conditions, invasive devices, and prolonged hospitalization and those who have received extended courses of antibiotics [12]. Gram-negative bacilli are responsible for many of nosocomial infections, including bloodstream infections, hospital-acquired pneumonia, urinary-tract infections, and increased mortality [7]. CRAB can survive on dry surfaces for long periods of time, which enables it to contaminate the hospital environment. *Klebsiella pneumoniae* can cause severe community-acquired infections in relatively healthy individuals [7]. According to Lansbury et al., *Pseudomonas aeruginosa* is the second most frequently detected pathogen in COVID-19 patients [13]. The rates of co-infection and secondary infection vary depending on the geographic region [14]. For example, in Iran, the leading cause of secondary bacterial infections was *Klebsiella* spp., followed by methicillin-sensitive *Staphylococcus aureus* (MSSA) and *Escherichia coli* [15], whereas in India, the most frequent isolate was *Escherichia coli*, followed by *Pseudomonas aeruginosa* and *Klebsiella* spp. [16].

Recent reports emphasize that, in patients with suspected or confirmed bacterial co-infection, early onset of antimicrobial treatment is crucial [5]. The widely used corticosteroid treatment may increase the risk and severity of secondary bacterial infections in patients with COVID-19. Therefore, in patients with a rapid decline in clinical condition, comprehensive evaluation for bacterial co-infection, as well as early empiric antimicrobial therapy, is mandatory [17]. 

Most of the patients hospitalized for COVID-19 received empiric antibiotic treatment, as noted by various studies [18]. It was observed that the levels of inflammatory serological markers such as C-reactive protein and procalcitonin, classically associated with bacterial infections, may also rise in COVID-19-affected individuals in the absence of bacterial co-infections [19,20]. This makes it difficult to distinguish between COVID-19 patients who may or may not need antibiotic treatment, based on routine laboratory data, before microbiological diagnostic becomes available. 

The main objective of this study was to evaluate the prevalence of secondary bacterial infections and the associated antibiotic resistance in COVID-19 patients.

## 2. Materials and Methods

This single-center retrospective, descriptive study included COVID-19 critical patients admitted in the COVID-19 Medical Support Unit, part of the Emergency Clinical Hospital Târgu Mures, between 1 August 2020 and 31 January 2021. The unit only admitted patients who needed intensive care treatment. 

COVID-19 Medical Support Unit Târgu Mures was a modular hospital intended for the treatment of critically ill COVID-19 patients, amid the healthcare system particularities of the first year of the pandemic. The necessity for building this modular hospital emerged after five months of pandemic, based on the increased number of COVID-19 cases in the county and nationwide, and the limited possibility of admission in the two preexistent Intensive Care Units in the county, which also had to ensure the treatment of non-COVID-19 medical emergencies. The Unit was organized in the location of a former sports facility, partitioned into multiple compartments, each having one intensive care bed with the specific equipment. Organ support capability was available as invasive and non-invasive ventilation, inotrope support, and tracheostomy. However, compartments were not entirely sealed at the ceiling level, and terminal disinfection by nebulization between patients could not be performed. Due to capacity constraints and to prevent accidental transmission of SARS-CoV-2 infection to non-COVID-19 patients necessitating ICU therapy, positive RT-PCR detection of SARS-CoV-2 was mandatory for patient admission to the Medical Support Unit Târgu Mures. Patients without RT-PCR confirmation of COVID-19 were treated in other medical care units, such as the Infectious Disease or Internal Medicine Clinics.

Inclusion criteria were as follows: patients with positive real-time reverse transcription polymerase chain reaction (RT-PCR) for detection of SARS-CoV-2 from a nasopharyngeal swab, severe clinical forms of the disease, thoracic computer tomography that described >70% ground glass opacities in both lungs, severe acute respiratory failure or other organ failures, admission in the ICU, and necessity of invasive or non-invasive mechanical ventilation. Exclusion criteria were as follows: age under 18, RT-PCR test for SARS-CoV-2 not performed or with a negative result, and non-ICU patients.

Knowing the increased risk of secondary bacterial infections in patients admitted to the ICU, the Carmeli score was calculated upon admission in order to identify the patients who were most susceptible to secondary bacterial infections and those who were probably colonized with opportunistic bacteria prior to the admission in the ICU. In the estimation of the Carmeli score, 1 point is attributed to patients under 65 years, without comorbidities, without previous antibiotic treatment, and with no contact with healthcare system; 2 points for patients over 65 years or with comorbidities, previous antibiotic treatment, or contact with the healthcare system; and 3 points for patients receiving invasive procedures or with immunocompromised status. Score 1 means low risk of colonization; score 2 means high risk of secondary bacterial infection associated to medical care; and score 3 means high risk of nosocomial infection with MDR pathogens.

Blood tests, including routine clinical chemistry, complete blood count, and other markers such as ferritin, C-reactive protein, procalcitonin, and D-dimers, were performed in an ISO 15189 [21] accredited laboratory at the Emergency Clinical Hospital Targu Mures. 

Blood cultures, endotracheal aspirate specimens, and stool and urine samples for the detection of secondary bacterial infections were collected upon admission in the COVID-19 medical care unit for all patients, repeated immediately after intubation and every 3–5 days thereafter. The specimens were inoculated on specific culture media to support the bacterial growth. After incubation, the microorganisms were identified using Gram staining and biochemical tests, completed with automated method Vitek 2 Compact when required. Antimicrobial susceptibility testing was performed using the disc diffusion method, completed with the evaluation of minimum inhibitory concentration (MIC) when needed, according to the EUCAST standard [22].

This study was performed in accordance with the Declaration of Helsinki, after approval of the Institutional Ethics Committee number 7526/12.03.2021. 

### Statistical Analysis

Data were extracted from electronic medical records using a standardized data collection form. Clinical and demographic data retrieved included age, sex, and comorbidities. Variables were centralized in Microsoft Excel (Microsoft 365, version 2407) spreadsheets. Statistical analysis was performed in the R statistical environment (version 4.3.3, cran.r-project.org, accessed on 15 April 2024). A threshold of 0.05 was considered for statistical significance. The normality of the distribution was assessed using the Shapiro–Wilk test. 

## 3. Results

Of a total of 243 Caucasian patients admitted to the COVID-19 Medical Support Unit of Târgu Mures between 1 August 2020 and 31 January 2021, 59 patients (24.3%) developed secondary infections (Table 1). 

All admitted patients had at least one comorbidity. Moreover, most of them presented multiple underlying diseases: 34 (14%) had obesity, diabetes, and cardiovascular disease; 35 (14.4%) had obesity and diabetes; and 96 (39.5%) had diabetes and cardiovascular disease. The prevalence of COPD among patients who developed secondary bacterial infections was 2.5 times higher than in patients without infectious complications (Table 1). 

The mean duration of hospitalization was higher in patients with secondary infections than in those without infectious complications (*p* = 0.006). The mean duration of invasive mechanical ventilation in COVID-19 patients with co-infections was 6.4 days (interval: 1–17 days). Non-invasive mechanical ventilation in COVID-19 patients with co-infections was needed for an average of 3.1 days (interval: 1–17 days). The mean length of stay in the ICU was 10 days (interval: 1–18 days).

Many of the patients with secondary infections were transferred from other hospitals (38 of 59 patients, 64.4%). Eleven (18.6%) of them had positive cultures for bacterial or fungal pathogens from samples collected within the first 48 h from admission. Early sample positivity is indicative of a secondary bacterial infection present upon admission and not a nosocomial infection acquired from the unit. Of the 11 patients, 1 had positive endotracheal aspirate positive for multidrug-resistant (MDR) *Klebsiella pneumoniae*; the others had positive cultures for *Enterococcus* or *Candida* spp. susceptible to common antimicrobial agents. We did not observe any statistically significant difference regarding the frequency of secondary infections between patients from our center and those transferred from other county hospitals (OR = 0.83, CI 95%: 0.43–1.63).

The Carmeli score was similar in patients with secondary infections and in those without bacterial or fungal infections (*p* = 1). In each of the two categories, about 30% of patients had a Carmeli score of 3, and none had a score equal to 1. Unsurprisingly, all patients had prior contact with healthcare facilities. Up to one third of the patients in the COVID-19 Support Unit had invasive interventions performed prior to admission. 

A total of 90 bacterial and fungal pathogens were identified in 72 biological specimens from the 59 patients: 25 blood cultures (34.7%), 38 endotracheal aspirates (52.8%), 5 urine cultures (6.9%), 3 stool cultures (4.2%), and 1 catheter tip (1.4%). Of the 25 blood cultures, 19 were positive upon first sampling, and 6 were positive upon second sampling (of which 4 within the same day and 2 after 2 and 13 days, respectively). 

Endotracheal aspirate samples become positive, on average, after 4 days from intubation. In 25 patients, the first endotracheal aspirate sample was positive; in 13 patients, the second sample was positive, collected after 6.1 days on average (interval 1–11 days). 

Microbiological results (identification and the antibiotic susceptibility testing results of the isolates) were available in an average of 4 days after sampling. In 28 (47.5%) cases, positive microbiological results were only available after the death of the patient.

Gram-negative bacteria were isolated from 55 (76.4%) samples, whereas Gram-positive bacteria were identified in 27 samples and fungi in 5 samples (Table 2). Bacterial co-infections with two different strains of Gram-negative bacteria were observed in 3 samples: two endotracheal aspirates (positive for *K. pneumoniae* ESBL, CPE + *A. baumannii* MDR, and *E. cloacae* + *M. morganii*, respectively) and one blood culture (positive for *K. pneumoniae* ESBL, CPE + *A. baumannii* MDR). 

The results of antimicrobial susceptibility testing showed a high-level resistance of MDR *Acinetobacter baumannii*. Among these strains, 14 (87.5%) were susceptible to colistin, 3 (19%) to amikacin, and 1 (6%) to trimethoprim/sulfamethoxazole. MDR *A. baumannii* strains were resistant to all the other tested antibiotics (including meropenem and imipenem).

Of the *Klebsiella pneumoniae* ESBL, CPE strains, 3 (30%) were susceptible to colistin, and 3 (30%) were susceptible to trimethoprim sulfamethoxazole. All *Klebsiella pneumoniae* CPE strains were susceptible to gentamycin and colistin. We found one single strain of *Klebsiella pneumoniae* without CPE or ESBL and CPE.

All four *Stenotrophomonas maltophilia* strains were susceptible to trimethoprim/sulfamethoxazole.

*Providencia stuartii* was resistant to all tested antibiotics, but it was not tested to colistin due to its intrinsic resistance and to imipenem due to its intrinsically low activity on this species. The patient died before the results of blood cultures became available.

*Pseudomonas aeruginosa* strains were susceptible to amikacin, piperacillin/tazobactam, and ceftazidime.

*Enterococcus* strains were susceptible to linezolid, teicoplanin, and vancomycin; one strain (7.1%) was resistant to vancomycin.

Although *Staphylococcus aureus* MRSA is known as a common secondary infection agent, we only identified 3 isolates, which were susceptible to vancomycin, linezolid, and teicoplanin.

The mortality rate in the COVID-19 Medical Support Unit Tirgu Mures was 84.4%, with 205 of 243 patients losing their lives due to COVID-19 and decompensation of underlying conditions during the 6 months of this study. Mortality in patients with secondary bacterial infection was even higher (93%, OR = 3.11, CI 95%: 1.04–12.60), regardless of the type of secondary infection (bacterial or fungal) or the site of infection. Among patients with secondary bacterial infections, at the end of this study, 4 out of 59 patients survived. 

## 4. Discussion

Our results describe the epidemiology of microbial infections associated with SARS-CoV-2 pneumonia in patients admitted to a support ICU organized for temporary function in conjunction with a tertiary-grade, emergency clinical hospital during the first stages of the COVID-19 pandemic. 

In our country, similarly with situations in other regions of the world, the healthcare system was overwhelmed by the high number of COVID-19 cases of various severities and suffered from recurrent interruptions in medicines and disinfectant supply chains, shortage of trained personnel, human resource burnout, and distress associated with the limited knowledge of the new virus biology and treatment possibilities [23,24].

The COVID-19 medical care unit from Târgu Mures was a brand-new facility that had not been used for other medical purposes prior to the admission of COVID-19 critically ill patients; the infrastructure and equipment were new, with low risk of prior colonization with MDR bacteria. Nevertheless, the secondary bacterial infections started to occur within one month from its opening due to the limitation of possibilities to perform disinfection through nebulization and isolation of patients. The facility was organized into 40 areas with intensive care unit hospital beds, separated by PVC walls, but with no ceiling, so the toxic vapors could have spread to the other stalls that had patients in them if disinfection by nebulization was performed. Although environmental cleaning can be performed in various manners, the most feasible for low resource healthcare facilities remains manual cleaning, as studies reported that a contaminated environment is a significant source of nosocomial infections [25]. In our COVID-19 medical care unit, surface sanitizer and UV lamps were used for disinfection, with limited results. Secondary bacterial infections inevitably emerged, with a prevalence similar to that from other intensive care units but with different etiology.

Patients admitted to other medical wards were transferred to the ICU due to aggravation of symptoms, severe respiratory failure, ARDS, and decompensation of underlying diseases, whereas some of them were directly admitted to the ICU because they had severe clinical forms of disease at presentation in the emergency departments from our hospital or from other hospitals in the country. Since we screened all patients upon their admission to the ICU, we could identify those who presented with secondary bacterial infections, with positive culture samples in the first 48 h from admission, and we isolated them. In those cases, the secondary bacterial infections probably favored the aggravation of symptoms and the necessity of intensive care treatment. In our group, secondary bacterial infections affected male patients more than females, and at a younger age, similarly with the findings reported by Graselli et al. [26].

In our intensive care unit, patients were vulnerable to secondary bacterial infections due to the multiple vascular access, immunosuppression, corticotherapy, several comorbidities, and multiple hospitalization episodes prior to the admission in the ICU, which can lead to colonization with MDR pathogens. Thus, they required broad spectrum antibiotics before the laboratory confirmation of secondary bacterial infections. Knowledge of the strains isolated in the COVID-19 Medical Care Unit and their susceptibility profile leads to the administration of empirical broad spectrum antibiotic treatment in these patients. Knowledge of the patients’ prior antibiotic exposure or history of severe infections prior to admission is necessary for the establishment of proper antibiotic treatment, having in mind the prevalence of infections with MDR pathogens and the concerning increase in strains resistant to antimicrobials.

Complex evaluation of critically ill patients is needed, and even more so in times of pandemic, when the quality of medical care assistance can decrease due to the overload of medical services, overcrowded wards, lack of sufficient medical staff, the burnout syndrome installing with a concerningly increased rate, and the high workload making clinical attendants vulnerable to errors. Almost all patients were screened upon admission: blood culture, endotracheal aspirate, and urine culture were sampled, and then repeated 3–5 days thereafter, which leads to the increased identification and report of secondary bacterial and fungal infections associated to medical care in the ICU despite the fact that all of the patients received empirical antibiotic treatment since their admission. As culture results were available postmortem in about half of the cases, those patients did not receive antibiotic treatment according to the antimicrobial susceptibility testing. 

We were able to identify secondary bacterial infections thanks to ongoing microbiological surveillance of samples from hospitalized patients, even though the first cultures were negative. Second blood cultures were positive in 24% of the cases, and second endotracheal aspirates were positive in 34% of the cases. These cases would have been overlooked if the samplings were not repeated.

The Carmeli score proved its predictive value in the assessment of patients with possible colonization or secondary bacterial infection upon admission. We expected that secondary bacterial infections with MDR pathogens would emerge in patients with score 3, in particular those with neoplasms, invasive procedures, or immunodeficiency. However, we identified most of the secondary bacterial infections with MDR pathogens in patients with score 2, i.e., those over 65 years, with comorbidities, prior antibiotic treatments, or contact with the healthcare system. Although patients with neoplasms, who are immunocompromised, or with invasive procedures prior to admission are the most vulnerable to secondary bacterial infections with MDR pathogens, they only represented a small fraction of our study group. The most affected patients were those with cardiovascular disease, obesity, or diabetes. Since the mortality rate was also high among patients without secondary bacterial infections, it is difficult to estimate if the outcome would have been different in the absence of secondary bacterial infections.

The mean time of invasive mechanical ventilation in patients with secondary bacterial infection was 6.4 days, as most patients died shortly after the aggravation of ARDS. Patients admitted in the ICU required multiple types of ventilation, and although they were admitted with non-invasive ventilation oxygen therapy, they required invasive mechanical ventilation within 1–3 days, meaning that the progression of the disease was rapid and the outcome unfavorable despite the specific therapy. The transfer of patients into the ICU should be performed prior to the onset of ARDS. 

The median length of stay in the ICU for patients with secondary bacterial infections was 10 days, longer than the one reported by Grasselli et al. [26] in COVID-19 patients. The mean duration of invasive mechanical ventilation in our COVID-19 patients with co-infections was 6.4 days, shorter than the period of 37 days reported by Buehler et al. [27].

In this study, we identified 27 cases of secondary infection associated with medical care, representing 11% of the cases admitted in the Support Unit during the period of this study. The particularity of our cohort consisted of the fact that most patients were transferred from other hospitals in the region only when their medical condition became critical, which partly explains the high mortality that we encountered. Another reason for the high prevalence of associated microbial infections may be the reluctance of patients with infection symptoms to address themselves in healthcare units during the pandemic, an aspect also observed by Zhu et al. [23]. 

Feng Y. and colleagues reported a multi-center study on COVID-19 of various severities. The patients were divided into 3 groups: moderately ill, severely ill, and critically ill. The authors found that the highest percentage of bacterial co-infection (34.5%) was identified in the critically ill patients compared with 3.9% in the moderately ill group and 8.3% in the severely ill group. A concerning aspect is that the higher rate of co-infections in critical patients occurred despite the fact that the majority received broad spectrum antibiotics [28].

Garcia-Vidal et al. reported a low rate of secondary bacterial and fungal infections in their cohort of 989 patients, although they only sampled 27% blood cultures and 13% endotracheal aspirate. In their study, they included 4% (*n* = 44) patients with severe clinical forms of COVID-19 admitted in the ICU, of which 56.8% (*n* = 25) developed secondary bacterial infections [29]. In comparison, we observed a lower prevalence (24.3%) of secondary infections in our cohort.

Pasero et al. published a meta-analysis on critically ill patients admitted with COVID-19 who developed infections caused by multidrug-resistant bacteria. In their analysis, they included 246 articles and found a 30% to 50% incidence of MDR microorganisms causing secondary bacterial infections. Gram-negative bacteria, predominantly CPE and *Pseudomonas aeruginosa*, were the most frequently involved [30].

The prevalence of secondary bacterial infections in the COVID-19 Medical Care Unit from Târgu Mures was similar to the prevalence of nosocomial infections in the ICU reported by Louis et al. [31] prior to the pandemic, meaning that attention to the possibility of co-infection occurrence was an important factor in the prevention of a rise in healthcare-associated infections, despite all difficulties encountered at that time. The prevalence of secondary bacterial infections in COVID-19 patients in this study was similar to the prevalence rate observed in a non-COVID-19 ICU.

The mortality rate was high in these patients due to secondary bacterial infections. They developed sepsis and multiple organ failure that did not respond to usual treatment. The management of previous comorbidities was also difficult. The mortality rate was high among patients who did not have secondary bacterial infections as well due to developed complications. 

In the year 2020, studies suggested that secondary bacterial infections were not common among patients with COVID-19. In 2021, numerous reports showed an increased incidence of secondary bacterial infections worldwide, especially in critically ill patients, but with different etiologies and different profiles of resistance to the antibiotics [3,32,33,34,35,36].

We had similar findings, except for the *Klebsiella* strains, which were CPE or BLSE in our case. We found very few strains susceptible to empiric antibiotics.

The rising incidence of secondary bacterial infections caused by MDR pathogens with resistance profiles differing from the pre-pandemic era, such as strains susceptible to trimetoprim/sulfametoxazol but resistant to carbapenems, fluoroquinolones, or colistin, was probably due to the widespread usage of broad spectrum antibiotics during the pandemic.

Several studies found that the development of secondary infections in COVID-19 patients can be predicted by various routine laboratory parameters. Leukocyte count was associated with blood culture positivity in COVID-19 patients in the report from Rebold et al. [37]. C-reactive protein was retained in the model of Schinkel et al., whereas leukocytes, neutrophils, and lymphocytes also had good feature importances among predictors of positive blood culture [38]. Age and procalcitonin were found as predictors by Murri et al. [39]. Tanzarella et al. identified neutrophils, C-reactive protein, and procalcitonin among the predictors of bacterial pneumonia in COVID-19 patients [40]. 

In our cohort, we observed that secondary infections were poorly associated with laboratory variables such as white blood cell count, C-reactive protein, procalcitonin, and ferritin. However, patients with COPD were more likely to develop secondary infections. 

We acknowledge several limitations of this study. First, we only used cultural methods to detect secondary pathogens. Culture-independent techniques such as PCR, targeted or multiplex, and next-generation sequencing, which are highly sensitive in the identification of potential pathogens, were not available in our laboratory. In consequence, some of the more fastidious pathogens may have remained undetected. Also, as in the case of other centers [41], some patients received antibiotic treatment even in the absence of confirmed bacterial co-infections. It is possible that some of the patients were falsely classified as not having secondary infections because of the microbial growth inhibition in clinical samples. We were not able to identify viral co-infections, although, recently, Alosaimi et al. reported a rate of co-infection with influenza AH1N1 reaching 64% [42]. Testing of other possible pathogens is important and should include viruses with similar manifestations and clinical characteristics, especially the ones with potential severe evolution. 

## 5. Conclusions

The prevalence of secondary bacterial infection associated to medical care represents a concern and needs particular attention from medical personnel even in the time of a pandemic. In our cohort, nosocomial infections were frequently produced by multidrug-resistant organisms and were associated with a high rate of mortality. 

This study emphasizes the geographical particularities of co-infections and secondary infections in critically ill COVID-19 patients. Despite COVID-19 being a well-characterized and widely studied pathology, small geographical regions faced particular challenges in disease management, and co-infections and secondary infections occurred even in newly organized facilities.

## Figures and Tables

**Table 1:** Characteristics of COVID-19 patients, in association with the presence of secondary infections.

Characteristic	Entire Group	Without Bacterial Infections	With Bacterial Infections	*p*-Value	N
	N = 243	N = 184	N = 59		
Age	70.0 [63.0; 77.0]	71.0 [62.0; 77.0]	70.0 [64.0; 76.0]	0.901	243
Sex:				0.58	243
F	96 (39.5%)	75 (40.8%)	21 (35.6%)		
M	147 (60.5%)	109 (59.2%)	38 (64.4%)		
White blood cell count, ×10^3^/µL	13.6 [9.8; 17.3]	13.2 [9.8; 17.5]	13.8 [9.9; 16.7]	0.911	240
Lactate dehydrogenase, U/L	590 [450; 786]	600 [452; 781]	570 [466; 807]	0.956	165
C-reactive protein, mg/L	126 [45.4; 194]	133 [53.7; 197]	113 [32.5; 190]	0.153	158
Procalcitonin, µg/L	0.84 [0.20; 2.95]	0.59 [0.18; 2.92]	1.27 [0.27; 2.95]	0.342	138
Ferritin, µg/L	1393 [834; 2462]	1321 [822; 2394]	1526 [916; 2768]	0.256	145
D-dimers, µg/L	1379 [672; 4271]	1156 [580; 2257]	1583 [980; 7856]	0.084	59
IL-6, ng/L	49.8 [23.8; 101]	50.5 [27.3; 101]	37.7 [18.8; 88.2]	0.833	45
Facial mask O_2_ therapy	22 (9.05%)	20 (10.9%)	2 (3.39%)	0.138	243
Non-invasive ventilation/CPAP	154 (63.4%)	114 (62.0%)	40 (67.8%)	0.513	243
Mechanical ventilation	67 (27.6%)	50 (27.2%)	17 (28.8%)	0.938	243
Carmeli score:				1	220
2	153 (69.5%)	113 (69.3%)	40 (70.2%)		
3	67 (30.5%)	50 (30.7%)	17 (29.8%)		
Deceased	205 (84.4%)	150 (81.5%)	55 (93.2%)	0.038	243
Hospitalization duration, days	8.00 [5.00; 12.0]	8.00 [4.75; 11.2]	10.0 [6.50; 13.0]	0.006	243
Comorbidities					
Obesity	74 (30.5%)	56 (30.4%)	18 (30.5%)	1	243
Diabetes mellitus	100 (41.2%)	72 (39.1%)	28 (47.5%)	0.328	243
Arterial hypertension	215 (88.5%)	160 (87.0%)	55 (93.2%)	0.282	243
Heart failure	154 (63.4%)	116 (63.0%)	38 (64.4%)	0.973	243
Liver pathology	35 (14.4%)	29 (15.8%)	6 (10.2%)	0.395	243
COPD	29 (11.9%)	16 (8.70%)	13 (22.0%)	0.012	243
CKD	27 (11.1%)	20 (10.9%)	7 (11.9%)	1	243
Malignancies	19 (7.82%)	15 (8.15%)	4 (6.78%)	1	243
Asthma	12 (4.94%)	8 (4.35%)	4 (6.78%)	0.492	243

Values are presented as number (percentage) for categorical variables and median [inter-quartile range] for continuous variables. N is the number of records available for each variable. F = female; M = male; IL-6 = interleukin 6, CPAP = continuous positive airway pressure; COPD = chronic obstructive pulmonary disease; CKD = chronic kidney disease.

**Table 2:** Bacterial isolates found in clinical samples of COVID-19 patients.

Pathogen	Number of Isolates, *n* (%)	Particular Resistance Phenotypes, *n* (%)
Gram-negative bacteria		
*Acinetobacter baumannii*	28 (31.1%)	MDR: 16 (57%)
*Klebsiella pneumoniae*	17 (18.9%)	CPE: 6 (35.3%) CPE and ESBL: 10 (58.8%)
*Pseudomonas aeruginosa*	4 (4.4%)	
*Stenotrophomonas maltophilia*	4 (4.4%)	
*Escherichia coli*	2 (2.2%)	
*Providencia stuartii*	1 (1.1%)	CPE and ESBL: 1 (100%)
*Morganella morganii*	1 (1.1%)	
*Enterobacter cloacae*	1 (1.1%)	
Gram-positive bacteria		
*Enterococcus faecium*	10 (11.1%)	
*Enterococcus faecalis*	4 (4.4%)	
*Staphylococcus aureus*	3 (3.3%)	MRSA: 1 (33%)
*Staphylococcus epidermidis*	2 (2.2%)	MRSE: 1 (50%)
*Staphylococcus haemolyticus*	1 (1.2%)	MRS: 1 (100%)
*Enterococcus galinarum/casseliflavus*	2 (2.2%)	
*Corynebacterium striatum*	2 (2.2%)	
*Clostridioides difficile*	3 (3.3%)	

## Data Availability

The data presented in this study are available on request from the corresponding author due to the confidential nature of medical information.
